# Prediction of Patients With Anaplastic Thyroid Carcinoma With Bone Metastasis: A Population-Based Study

**DOI:** 10.1155/ije/2209918

**Published:** 2025-06-23

**Authors:** Yisong Yao, Guibin Zheng, Xi Chen, Yaqi Wang, Congxian Lu, Jiaxuan Li, Ting Yuan, Caiyu Sun, Yakui Mou, Yumei Li, Xicheng Song

**Affiliations:** ^1^Department of Otorhinolaryngology, Head and Neck Surgery, Yantai Yuhuangding Hospital, Qingdao University, Yantai, China; ^2^Shandong Provincial Key Laboratory of Neuroimmune Interaction and Regulation, Yantai, China; ^3^Shandong Provincial Clinical Research Center for Otorhinolaryngologic Diseases, Yantai, China; ^4^Yantai Key Laboratory of Otorhinolaryngologic Diseases, Yantai, China; ^5^Department of Thyroid Surgery, The Affiliated Yantai Yuhuangding Hospital of Qingdao University, Yantai, China

**Keywords:** anaplastic thyroid carcinoma, bone metastasis, machine learning, prediction

## Abstract

**Background:** Bone metastasis (BM) is a significant risk factor for the survival and prognosis of patients with anaplastic thyroid carcinoma (ATC). The aim of this study was to predict BM in patients with ATC.

**Methods:** Demographic and clinicopathological data of patients with ATC were extracted from the Surveillance, Epidemiology, and End Results database between 2010 and 2020. Logistic regression (LR) was used to identify the linear influencing factors for BM. We developed prediction models for BM using six machine learning models: support vector machine (SVM), LR, adaptive boosting (AD), decision tree (DT), eXtreme Gradient Boosting (XGB), and random forest (RF). The area under the receiver operating characteristic curve (AUC) values, accuracy, recall rate, precision, *F*1 scores, calibration curves, and precision-recall curves were used to determine the best model and evaluate its effectiveness. The SHapley Additive exPlanations algorithm was used to reveal the interpretability of the prediction model.

**Results:** This study included 781 patients with ATC, of whom 78 (9.99%) patients occurred BM and 703 (90.01%) patients were free of BM. The XGB model significantly outperformed the other models, with the highest *F*1 (0.897), accuracy (0.878), precision (0.924), recall (0.900), and AUC (0.897) values. The results of the LR model showed that age, gender, lung metastasis, and liver metastasis were linear influencing factors. According to XGB model, metropolitan area, median household income, N stage, and race were also strongly associated with BM among patients with ATC.

**Conclusion:** We explored influencing factors for BM and established a prediction model based on XGB that yielded excellent results in predicting BM in patients with ATC. This study provides a theoretical basis for early decision making in clinical practice.

## 1. Introduction

The incidence of thyroid cancer (TC) has increased dramatically worldwide in recent decades [1], making it the most common malignant tumor of the endocrine system [2]. Based on differences in tumor origin and differentiation, TC is mainly classified into papillary thyroid carcinoma (PTC), follicular thyroid carcinoma (FTC), medullary thyroid carcinoma (MTC), and anaplastic thyroid carcinoma (ATC) [1, 2]. Among them, ATC is a relatively rare malignant thyroid tumor, accounting for only 1% of all TCs [3]. However, because the majority of ATC is highly malignant and aggressive, surgery often fails to achieve satisfactory results, and chemoresistance frequently develops during clinical treatment. The long-term survival prognosis of patients with ATC is often poor [4, 5], with a median survival of only 5 months and a 1-year overall survival rate of only 20% [6].

Distant metastasis (DM) has been reported to occur in 50% of patients with ATC [7] and is characterized by extrathyroidal growth, vascular lymphatic infiltration, and distant organ invasion [8], while only 10% of patients with ATC have progression limited to the thyroid [9]. DM has long been recognized as the most important independent risk factor affecting the survival prognosis of patients [10], with a median survival of 171 days in ATC patients with DM, a 1-year overall survival rate of 15% [7], and 70% of patients dying within 4 years of diagnosis [11].

Bone is one of the most common sites of DM in patients with TC [8]. The incidence of bone metastasis (BM) is approximately 4% in patients with TC [12] and up to 5%–15% in patients with ATC [6]. Patients with TC with BM usually have a worse prognosis, with a 10-year survival rate as low as 27% [13]. In addition, 78% of patients develop severe clinical bone-related events (SREs), and more than 60% of SREs recur during the long-term course of ATC, often leading to a variety of symptoms, such as bone pain, limb movement limitation, and even pathological fracture, which greatly reduce patients' quality of life [14]. However, most BMs are asymptomatic and can only be detected in a few cases, such as when patients present with SREs or undergo unconventional whole-body bone scans [15], by which time the majority of patients with ATC have already progressed to an advanced stage or missed the optimal time for treatment [6]. Therefore, it is essential to explore the occurrence of BM in patients with ATC in a timely manner.

Most studies based on the Surveillance, Epidemiology, and End Results (SEER) database defined the study population as newly diagnosed TC or specific histological subtypes (e.g., differentiated thyroid cancer (DTC)) [15–18]. No study has yet focused on the development of BM in patients with ATC [19]. Therefore, this study focused on the development of BM in patients with ATC using the SEER program.

## 2. Methods

### 2.1. Study Design and Patients

This study included samples from the SEER program, which includes demographic, tumor pathology, and clinical treatment information for patients with cancers [20]. After obtaining a license and regulatory approval for the SEER database, we included data on patients with ATC between 2010 and 2020 because information on specific DM (such as BM) was not available until 2010 [21]. Additional information is available on the official SEER database website (https://seer.cancer.gov/about/).

The inclusion criteria were as follows: (1) year of diagnosis: 2010–2020; (2) histologically confirmed diagnosis of ATC according to the International Classification of Diseases of Oncology, 3rd Revision (ICD-O-3) histological codes, which were mainly 8020/3, 8021/3, 8022/2, 8022/3, 8023/3, 8030/3, 8031/3, 8032/3, 8032/3, 8033/3, 8034/3, 8035/3, and 8805/3; (3) patients with ATC with BM requiring confirmation by imaging and/or pathology; and (4) patients with ATC without brain metastasis. The exclusion criterion included lack of specific information. Ethical approval and informed consent were not required for this study because the SEER database does not release personally identifiable information.

Patients' data were downloaded from the “Incidence-SEER Research Data, 17 Registries, Nov 2022 Sub (2000–2020)” database using SEER∗Stat 8.4.2 software (released 8/14/2023). The AJCC 7th edition was released in 2009, so patients diagnosed between 2010 and 2020 were staged using AJCC 7th edition.

### 2.2. Variables and Endpoints

Variables extracted from the SEER database included age, gender, race, marital status, median household income, metropolitan area, grade, tumor size, T stage, N stage, liver metastasis, lung metastasis, laterality, and the number of tumors. The primary endpoint of this study was BM.

### 2.3. Data Preprocessing

Categorical variables underwent one-hot coding [22]. It is worth noting that the incidence of BM in patients with ATC was extremely low, indicating a highly imbalanced original dataset. Stratified sampling was used to divide the dataset into a training set (80%) and a test set (20%). To address the imbalance, we used oversampling to preprocess the raw data, analyzing changes in the dataset through a correlation matrix [23]. The Synthetic Minority Over-sampling Technique for Nominal (SMOTEN) used in this study is a standard approach for balancing class distribution in imbalanced datasets.

### 2.4. Linear Influencing Factor Selection

After univariate analysis, significant variables were subjected to multivariate logistic regression (LR) analyses to confirm linear influencing factors of ATC patients with BM.

### 2.5. Prediction Model Training and Validation

Considering the complex interactions between variables and the nonlinear relationships that may be overlooked by LR, we conducted ablation studies to determine the factors influencing the occurrence of BM in patients with ATC and the optimal model for predicting it. We conducted two models in turn. For the first model, we integrated four significant influencing factors suggested by the multivariate LR model. To maximize the incremental information included in the prediction model, for the second model, we integrated relevant variables from the SEER database into the model based on previous literature and clinical experience. The dataset was divided into a training set and a test set in a ratio of 8:2. The training set was subjected to analysis using six machine learning (ML) algorithms trained by 10-fold cross-validation to construct BM prediction models. These algorithms included support vector machine (SVM), LR, adaptive boosting (AD), decision tree (DT), eXtreme Gradient Boosting (XGB), and random forest (RF). Our literature review indicated that these ML models have been widely used in the clinical prediction of TC [24, 25]. The test set was used to assess the performance of these ML models. Accuracy, recall rate, area under the receiver operating characteristic (ROC) curve (AUC) value, precision, and the *F*1 score were used to evaluate these ML models. The methods used to compute these hyperparameters are presented in the following equations:

(1) $$\begin{array}{c} A\text{ccuracy}=\frac{\text{TP}+\text{TN}}{\text{TP}+\text{TN}+\text{FP}+\text{FN}},\\ R\text{ecall rate}=\frac{\text{TP}}{\text{TP}+\text{FN}},\\ \text{AUC}=\frac{\sum_{{\text{ins}}_{i}\varepsilon \text{pos}}{\text{rank}}_{{\text{ins}}_{i}}-\left(\left(M*\left(M+1\right)\right)/2\right)}{M*N},\\ P\text{recision}=\frac{\text{TP}}{\text{TP}+\text{FP}},\\ F1\text{ score}=\frac{2\text{TP}}{2\text{TP}+\text{FP}+\text{FN}}. \end{array}$$

We also used a calibration curve and a precision-recall (PR) curve to test the reliability of the prediction model. Additionally, we developed an interpretable model for ML based on the SHapley Additive exPlanations (SHAP) algorithm [26]. In this process, we visualized and interpreted 1 randomly selected patient and 20 randomly selected patients, respectively. The waterfall plot reveals how values are driven from the model's expected output values on the dataset to the model's predicted output values. The decision plot shows how SHAP values start at the base value and move to the top of the graph. Finally, partial dependence plots (PDPs) were applied for each feature's interpretability [27].

### 2.6. Statistical Analysis

Data were compiled and stored using Microsoft Excel spreadsheets (Microsoft Corp., Redmond, WA, USA). Categorical variables were presented as counts and percentages, while continuous variables were expressed as means ± standard deviation. Normal distribution was confirmed using the Shapiro–Wilk normality test. Differences between patients with and without BM were assessed using the *t*-test for continuous variables, and categorical variables were compared using the chi-squared or Fisher's exact test, as appropriate. The Kruskal–Wallis test was used for multiple categorical variables. Data analysis was conducted using SPSS 26.0 (IBM Corp., Armonk, NY, USA), Python 3.8.0 (https://www.python.org/), and SEER∗Stat (https://seer.cancer.gov/seerstat/) software.

## 3. Results

### 3.1. Characteristics of the Patients With ATC

A total of 781 patients with ATC were included in this study. Among them, BM occurred in 78 patients (9.99%) and 703 patients were free of BM at initial diagnosis (90.01%). Age, sex, T stage, N stage, lung metastasis, and liver metastasis were significantly different between the two groups (*p* < 0.05). The detailed information is shown in Table 1.

### 3.2. Linear Influencing Factor Selection

In the multivariate LR model, age, sex, liver metastasis, and lung metastasis were identified as independent predictors of BM in ATC. Further details can be found in Table S1.

### 3.3. Prediction Model Performance

We used six ML algorithms, including SVM, XGB, LR, AD, DT, and RF, to develop BM prediction models for patients with ATC. Optimal parameters were determined through a grid search. Firstly, we integrated age, gender, liver metastasis, and lung metastasis in ML models. The performance of the models was relatively average, with AUCs mostly ranging from 0.716 to 0.719 (Table S2).

**Figure 1:**
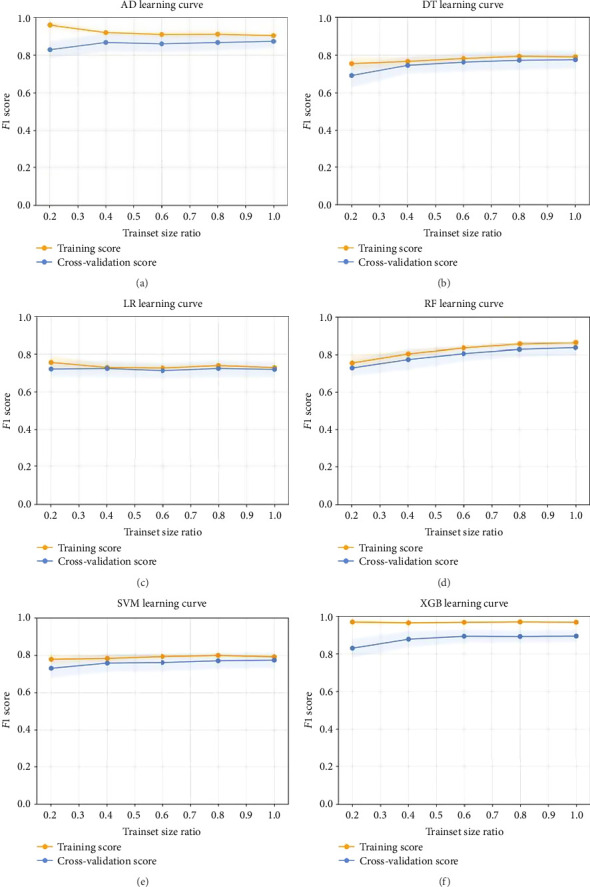
Learning curves for all prediction models with oversampling. Abbreviations: AD, adaptive boosting; DT, decision tree; RF, random forest; LR, logistic regression; SVM, support vector machine; XGB, eXtreme Gradient Boosting.

Then, a total of 14 variables were included in these models, including age, gender, race, marital status, median household income, metropolitan area, grade, tumor size, T stage, N stage, liver metastasis, lung metastasis, laterality, and the number of tumors. As the number of training set samples increased, the *F*1 scores for all prediction models exceeded 0.7, which indicated robust performance and demonstrated effective predictive capabilities with no apparent overfitting (Figure 1).

**Figure 2:**
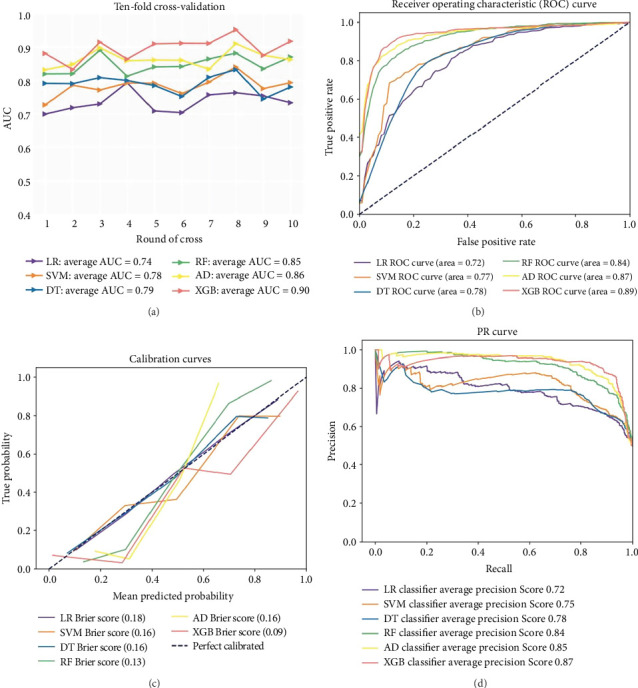
Ten-fold cross-validation for average AUC (a), receiver operating characteristic (ROC) curve (b), calibration curves (c), and precision-recall (PR) curve (d) for all prediction models with oversampling. Abbreviations: AD, adaptive boosting; DT, decision tree; RF, random forest; LR, logistic regression; SVM, support vector machine; XGB, eXtreme Gradient Boosting.

Ten-fold cross-validation was used to explore the average AUC of each ML model (Figures 2(a) and 2(b)). The XGB prediction model significantly outperformed the other models, achieving the highest average AUC (0.90). Additionally, we performed the *t*-test to compare the 10-fold cross-validation AUC between different models (Table 2). The XGB model was also better than others. More details about XGB are shown in Table S3.

A calibration curve was used to visualize the consistency between the predicted and observed variables in the six BM prediction models for patients with ATC (Figure 2(c)). On the curve, the 45-degree blue straight line represents the perfect match between the observed (*y*-axis) and predicted (*x*-axis) variables. A smaller distance between actual and gray straight line indicates a better outcome.  Figure 2(d) shows the PR curves for six ML models for predicting BM in patients with ATC. The XGB prediction model significantly outperformed the other models, achieving the highest *F*1 (0.897), accuracy (0.878), precision (0.924), recall (0.900), and AUC (0.897) values among all the models (Figure 2 and Table 3).

### 3.4. Prediction Model Interpretability

**Figure 3:**
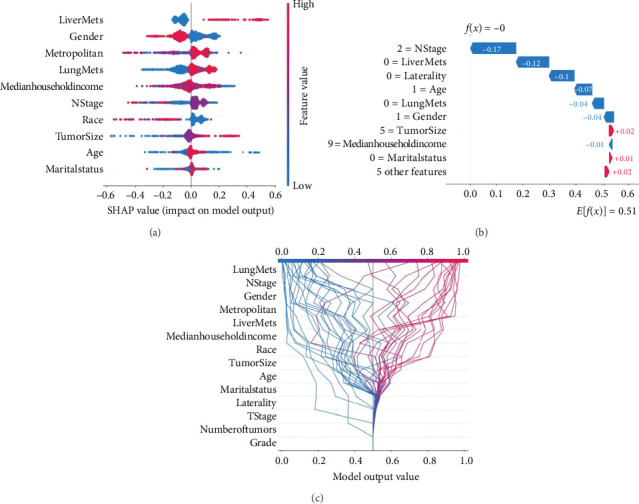
(a) Explanatory visualization of individuals' predictions. (b) Explanatory visualization of single individual's predictions. (c) Visual decision plot for multiple sample prediction.

Based on the SHAP plot of the XGB model, lung metastasis, liver metastasis, gender, metropolitan area, median household income, N stage, and race emerged as the most important factors influencing BM development in patients with ATC (Figure 3(a)). The plot illustrated the individual characteristics of patients with ATC who are at increased or decreased risk of developing BM. This information can be used for individualized healthcare planning with prospective implications based on the predicted XGB model.  Figure 3(b) shows a male ATC patient with a partner who was unlikely to develop BM.  Figure 3(c) displays a decision plot where each line represented a participant, and the SHAP value indicated the contribution of each feature to the final category. Finally, features' interpretability was shown as PDP in Figures S1–S7.

## 4. Discussion

Patients with ATC with BM usually have a poor prognosis. The insidious nature of early clinical symptoms and the complexity of diagnostic modalities have made recognizing BM at the initial diagnosis a long-standing clinical challenge [28]. This study aims for risk factor investigation and prediction model establishment for BM in patients with ATC.

Since LR can only identify limited linear relationship, this study firstly identified significant linear influencing factors affecting the occurrence of BM in patients with ATC by LR, including age, gender, liver metastasis, and lung metastasis. In addition, we performed ablation studies to explore all potential influencing factors associated with BM and the optimal prediction model. We found that the effect of the prediction model which included 14 variables based on XGB (AUC = 0.897) was significantly better than the effect of the prediction model built from four significant linear influencing factors (AUC = 0.718)). Therefore, we were able to determine that there were still variables whose effects were not observed by the LR model. The most effective algorithm, XGB, was selected to construct a clinically informed model to predict BM in patients with ATC. Through the comparison of SHAP algorithm, we identified significant associations between lung metastasis, liver metastasis, gender, metropolitan area, median household income, N stage, and race with BM in patients with ATC.

In this study, we found that the results of different ML models all showed that DMs in the lung and liver were risk factors for the development of BM in patients with ATC, which might suggest that those patients tend to have multiple DMs. According to Besic and Gazic [29], 84% of ATC cases had two or more metastatic sites. It was also noted that about 27.6% of patients were found to have multiple organ metastasis at the time of initial diagnosis, and solitary BMs were even rarer [19]. This metastasis pattern is not exclusive to BM in ATC, but also occurs in other cancers. For breast cancer, other distant organ metastasis can also indicate the possibility of BM [30]. Wang et al. also noted that both bone and lung metastases can be significant predictors of liver metastasis in patients with colorectal cancer [31]. For tumors that might be highly malignant, there could be a relationship between different distant organ metastases. It has been shown that the accumulation of senescent cells can expedite the development of a fused bone environment, leading to the recurrence and metastasis of dormant ATC, while DMs to other sites are occurring [32, 33]. Additionally, surgically induced physical damage and inflammation-induced chemical damage have been linked to metastatic growth at nonsurgical sites [34–36].

In addition to tumor clinical information, sociodemographic characteristics play an important role in predicting BM risk in patients with ATC. Relevant studies have revealed that although the incidence of TC is higher in women than in men [37], the rate of extrathyroidal tissue invasion, distal metastasis, recurrence, and mortality are higher in male patients than in women [38]. Our study also showed that men were a risk factor for BM. Previous studies might have overlooked the influence of metropolitan area on disease outcomes. Medical insurance measures in larger scale cities are more comprehensive, and a complete health insurance system can effectively improve the disease outcomes of patients with cancers [39]. In addition, larger scale cities might have more complete medical facilities with advanced technology, providing patients with more equal access to medical care and more timely medical screening and interventions. Household income may have the same mechanisms with city scale and medical insurance as disposable financial resources. Moreover, ethnicity can be a risk factor. Among patients with DTC, black patients had a greater risk of BM [40]. Similarly, in the population of breast cancer patients, outcomes for black patients were worse [41]. However, according to the results of our study, the predicted risk factor for BM of ATC is white ethnicity. This could be the result of the uneven distribution of respondents among different racial groups. Additionally, we also found that older patients were more likely to have BM. We speculated that it may be caused by physical development. Aging is accompanied by cell aging, including changes of protein, metabolism, and genome instability, which can be involved in the progression of tumors [42].

It is worth noting that lymph node metastasis also has a significant influence on the occurrence of BM in patients with ATC. Previous studies have highlighted the importance of lymph node metastasis in predicting BM in patients with TC with various pathological subtypes such as PTC, MTC, and FTC [43–45].

However, this study has certain limitations. First, the SEER database only provides cases with synchronous metastasis, and BM often occurs during the follow-up of patients with TC, which might lead to a large sample loss of patients with BM. This might consequently diminish the precision of this prediction model in real-world settings. Additionally, we did not perform validation against external databases, which might have affected our ability to prove the extensibility of this prediction model to some extent. Therefore, more clinical studies, especially randomized clinical trials, are needed in the future.

## 5. Conclusions

The present study developed a XGB-based prediction model for BM in patients with ATC, which was capable of outperforming other ML models. The results of the LR model showed that age, gender, lung metastasis, and liver metastasis were linear influencing factors. According to the XGB model, metropolitan area, median household income, N stage, and race were also strongly associated with BM among patients with ATC. This might facilitate personalized diagnosis and refine clinical decision making for BM in patients with ATC.

## Figures and Tables

**Table 1:** Characteristics of patients with ATC.

Factor	Bone metastasis		*X* ^2^	*p*
	No (*n* = 703)	Yes (*n* = 78)		
Age			1.637	< 0.05
20–44 years	10 (1.4%)	2 (2.6%)		
45–64 years	233 (33.1%)	30 (38.5%)		
≥ 65 years	460 (65.4%)	46 (59%)		
Race			1.971	0.578
White	556 (79.1%)	62 (79.5%)		
Black	54 (7.7%)	5 (6.4%)		
Other	91 (12.9%)	10 (12.8%)		
Unknown	2 (0.3%)	1 (1.3%)		
Sex			8.288	< 0.05
Male	290 (41.3%)	46 (59%)		
Female	413 (58.7%)	32 (41%)		
Marital status			1.641	0.440
Have no partner	287 (40.8%)	26 (33.3%)		
Have a partner	392 (55.8%)	49 (62.8%)		
Unknown	24 (3.4%)	3 (3.8%)		
Median household income			6.880	0.650
< $35,000	10 (1.4%)	0 (0%)		
$35,000–$39,999	9 (1.3%)	2 (2.6%)		
$40,000–$44,999	14 (2%)	1 (1.3%)		
$45,000–$49,999	30 (4.3%)	7 (9%)		
$50,000–$54,999	41 (5.8%)	4 (5.1%)		
$55,000–$59,999	54 (7.7%)	5 (6.4%)		
$60,000–$64,999	88 (12.5%)	10 (12.8%)		
$65,000–$69,999	92 (13.1%)	13 (16.7%)		
$70,000–$74,999	92 (13.1%)	8 (10.3%)		
$75,000+	273 (38.8%)	28 (35.9%)		
Metropolitan			5.755	0.218
Nonmetropolitan not adjacent to a metropolitan area	39 (5.5%)	5 (6.4%)		
Nonmetropolitan adjacent to a metropolitan area	45 (6.4%)	10 (12.8%)		
Metropolitan of < 250 K inhabitants	37 (5.3%)	5 (6.4%)		
Metropolitan > 250 K–1 M inhabitants	174 (24.8%)	14 (17.9%)		
Metropolitan > 1 M inhabitants	408 (58%)	44 (56.4%)		
Grade			0.452	0.501
Grade I	0 (0.0%)	0 (0.0%)		
Grade II	0 (0.0%)	0 (0.0%)		
Grade III	7 (1%)	2 (2.6%)		
Grade IV	696 (99%)	76 (97.4%)		
Laterality			0.155	0.925
Left	75 (10.7%)	8 (10.3%)		
Right	6 (0.9%)	1 (1.3%)		
Bilateral/NOS	622 (88.5%)	69 (88.5%)		
T Stage			11.227	< 0.05
T0	0 (0%)	1 (1.3%)		
T1	2 (0.3%)	0 (0%)		
T2	7 (1%)	0 (0%)		
T3	38 (5.4%)	2 (2.6%)		
T4	650 (92.5%)	75 (96.2%)		
Tx	6 (0.9%)	0 (0%)		
N stage			9.055	< 0.05
N0	239 (34%)	15 (19.2%)		
N1	399 (56.8%)	58 (74.4%)		
Nx	65 (9.2%)	5 (6.4%)		
Tumor size			9.689	0.085
< 10	3 (0.4%)	0 (0%)		
10–19	15 (2.1%)	2 (2.6%)		
20–29	21 (3%)	5 (6.4%)		
30–39	53 (7.5%)	3 (3.8%)		
≥ 40	510 (72.5%)	49 (62.8%)		
Unknown	101 (14.4%)	19 (24.4%)		
Liver metastasis			54.875	< 0.05
No	687 (97.7%)	62 (79.5%)		
Yes	16 (2.3%)	16 (20.5%)		
Lung metastasis			30.990	< 0.05
No	465 (66.1%)	26 (33.3%)		
Yes	238 (33.9%)	52 (66.7%)		
Number of tumors			0.478	0.569
1	666 (94.7%)	74 (94.9%)		
> 1	37 (5.3%)	4 (5.1%)		

**Table 2:** The *t*-test to compare the 10-fold cross-validation AUC between different models.

Models	*t* value	*p*
XGB compared to LR	−11.696967197853882	< 0.001
XGB compared to SVM	−10.772999784004162	< 0.001
XGB compared to DT	−9.86378420619267	< 0.001
XGB compared to RF	−9.646534116888832	< 0.001
XGB compared to AD	−3.537375803165431	0.006

Abbreviations: AD = adaptive boosting, AUC = area under the curve, DT = decision tree, LR = logistic regression, RF = random forest, SVM = support vector machine, and XGB = eXtreme Gradient Boosting.

**Table 3:** Performance comparison between prediction models based on different machine learning algorithms.

Models	AUC	Accuracy	Precision	Recall rate	*F*1 score
XGB	0.897	0.878	0.924	0.900	0.897
AD	0.861	0.897	0.817	0.853	0.861
SVM	0.865	0.846	0.889	0.867	0.864
LR	0.735	0.737	0.733	0.733	0.736
DT	0.790	0.789	0.790	0.788	0.790
RF	0.847	0.853	0.839	0.846	0.848

Abbreviations: AD = adaptive boosting, AUC = area under the curve, DT = decision tree, LR = logistic regression, RF = random forest, SVM = support vector machine, and XGB = eXtreme Gradient Boosting.

## Data Availability

The datasets generated and/or analyzed during the current study are available in the SEER database (https://seer.cancer.gov/). Further inquiries can be directed to the corresponding authors.

## Supporting Information

Table S1: multivariate logistic regression of factors related to distant metastasis—we identified that age, sex, liver metastasis, and lung metastasis were independent predictors of BM in ATC. Table S2: performance comparison between prediction models based on different machine learning algorithms including four linear influencing factors—we found that the performance of the models built by four linear significant factors was relatively average, with AUCs mostly ranging from 0.716 to 0.719. Table S3: detailed settings for the XGB model—we reported more details about XGB model. Figure S1: partial dependent plots of clinical characteristics “gender”; the shaded portion represents the confidence interval—we found that men were a risk factor for BM. Figure S2: partial dependent plots of clinical characteristics “liver metastasis”; the shaded portion represents the confidence interval—we found that liver metastasis was a risk factor for BM. Figure S3: partial dependent plots of clinical characteristics “lung metastasis”; the shaded portion represents the confidence interval—we found that individuals with lung metastasis had higher risk of BM. Figure S4: partial dependent plots of clinical characteristics “median household income”; the shaded portion represents the confidence interval—we found that patients with higher median household income had lower risk of BM. Figure S5: partial dependent plots of clinical characteristics “metropolitan”; the shaded portion represents the confidence interval—we found that patients from larger metropolitan had lower risk of BM. Figure S6: partial dependent plots of clinical characteristics “N stage”; the shaded portion represents the confidence interval—we found that patients with higher N stage had greater risk of BM. Figure S7: partial dependent plots of clinical characteristics “race”; the shaded portion represents the confidence interval—we found that black patients with ATC had greater risk of BM.
