# Supplementary material for: Prediction of Patients With Anaplastic Thyroid Carcinoma With Bone Metastasis: A Population-Based Study
Source: Int J Endocrinol. 2025 Jun 23;2025:2209918. doi: 10.1155/ije/2209918 (PMC12208758; doi:10.1155/ije/2209918)
Supplement: Supporting Information — Additional supporting information can be found online in the Supporting Information section. [file 2209918.f1.docx]

**Supplementary Tables**

**Table S1.** Multivariate logistic regression of factors related to distant metastasis.

|  | Multivariate logistic regression | |
| --- | --- | --- |
|  | OR(95%*CI*) | *P* |
| Age |  |  |
| 20–44 years | Ref |  |
| 45–64 years | 0.174 (0.050~0.600) | 0.006 |
| ≥ 65 years | 0.147 (0.044~0.492) | 0.002 |
| Sex | - | - |
| Male | Ref |  |
| Female | 0.519 (0.302~0.892) | 0.018 |
| Liver metastasis |  |  |
| No | Ref |  |
| Yes | 9.544 (4.239~21.484) | <0.001 |
| Lung metastasis |  |  |
| No | Ref |  |
| Yes | 2.899 (1.700~4.945) | <0.001 |

**Table S2.** Performance comparison between prediction models based on different machine learning algorithms including four linear influencing factors.

| Models | AUC | Accuracy | Precision | Recall rate | F1 score |
| --- | --- | --- | --- | --- | --- |
| XGB | 0.718 | 0.750 | 0.655 | 0.699 | 0.718 |
| AD | 0.717 | 0.746 | 0.658 | 0.699 | 0.717 |
| SVM | 0.719 | 0.750 | 0.656 | 0.700 | 0.719 |
| LR | 0.716 | 0.743 | 0.642 | 0.697 | 0.716 |
| DT | 0.717 | 0.746 | 0.659 | 0.700 | 0.717 |
| RF | 0.717 | 0.745 | 0.659 | 0.699 | 0.717 |

AD, adaptive boosting; AUC, area under the curve; DT, decision tree; LR, logistic regression; XGB, eXtreme Gradient Boosting; SVM, support vector machine; RF, random forest; AUC, the area under the curve.

**Table S3.** Detailed settings for the XGB model.

| Learning rate | Max depth | lambda | Min child weight | Subsample | Colsample bytree |
| --- | --- | --- | --- | --- | --- |
| 0.5 | 6 | 0 | 1 | 0.8 | 0.8 |

XGB, eXtreme Gradient Boosting.

**Supplementary Figures**


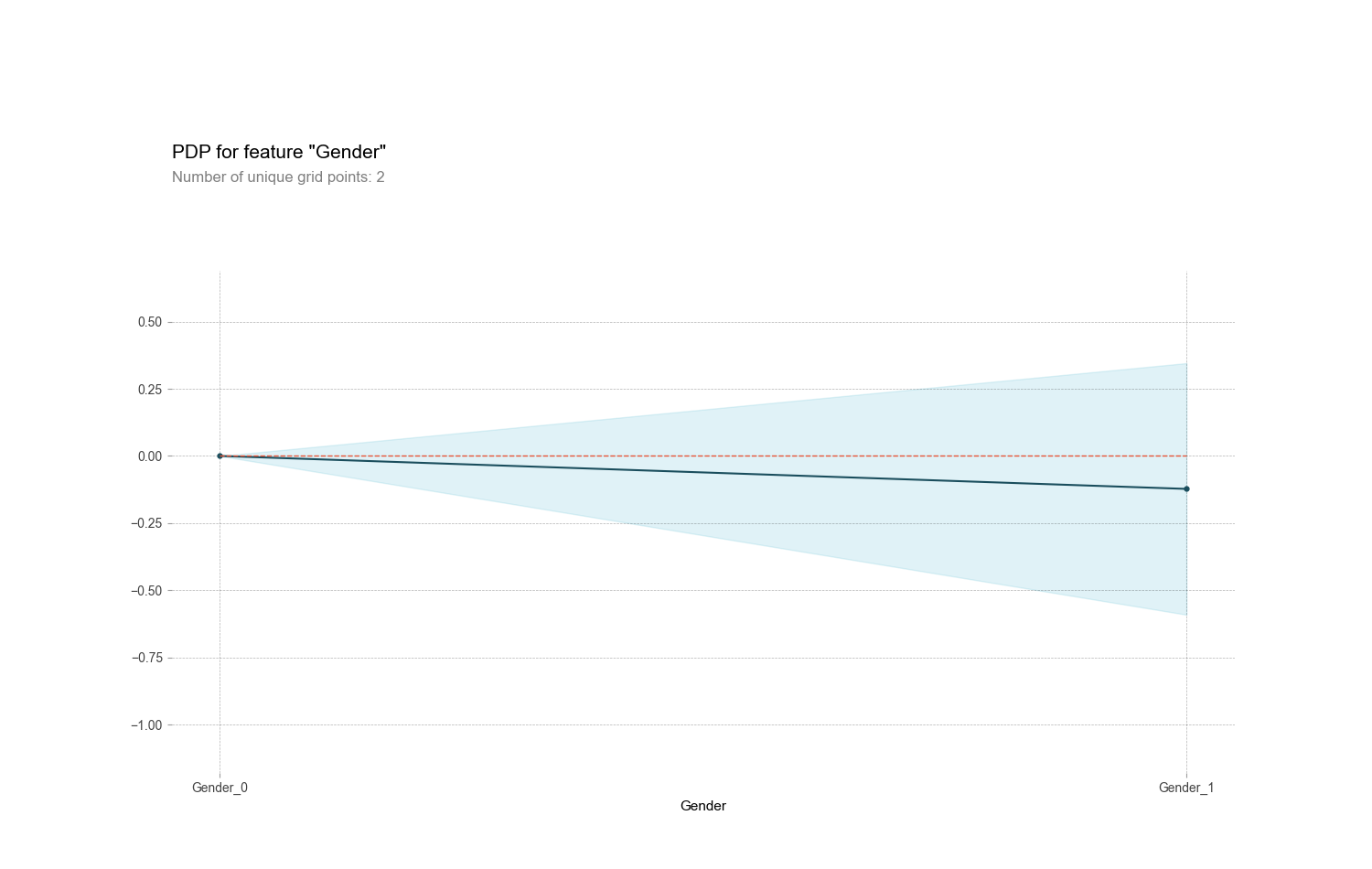


**Figure S1.** Partial dependent plots of clinical characteristics “gender”; the shaded portion represents the confidence interval.


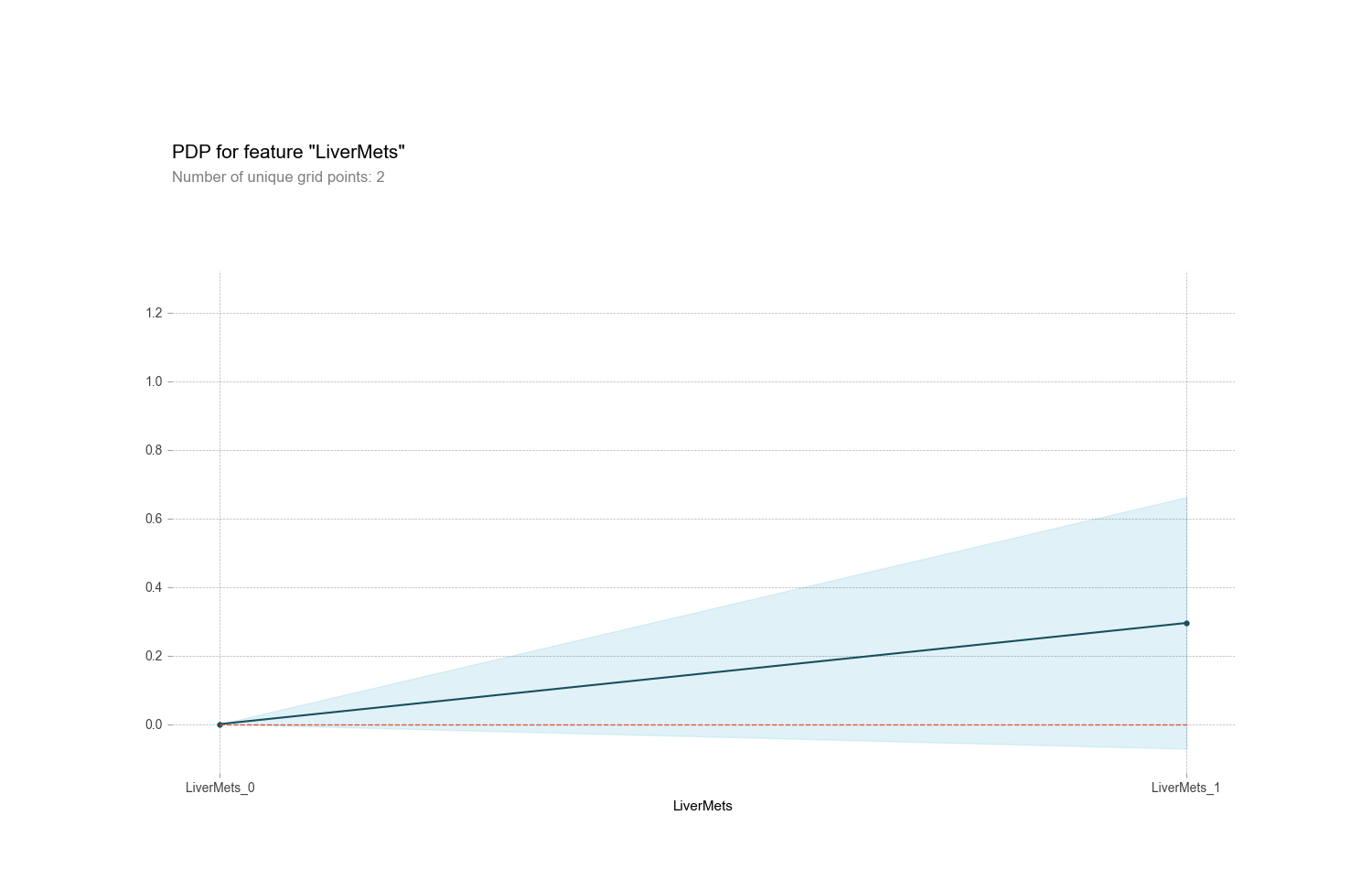


**Figure S2.** Partial dependent plots of clinical characteristics “liver metastasis”; the shaded portion represents the confidence interval.

**
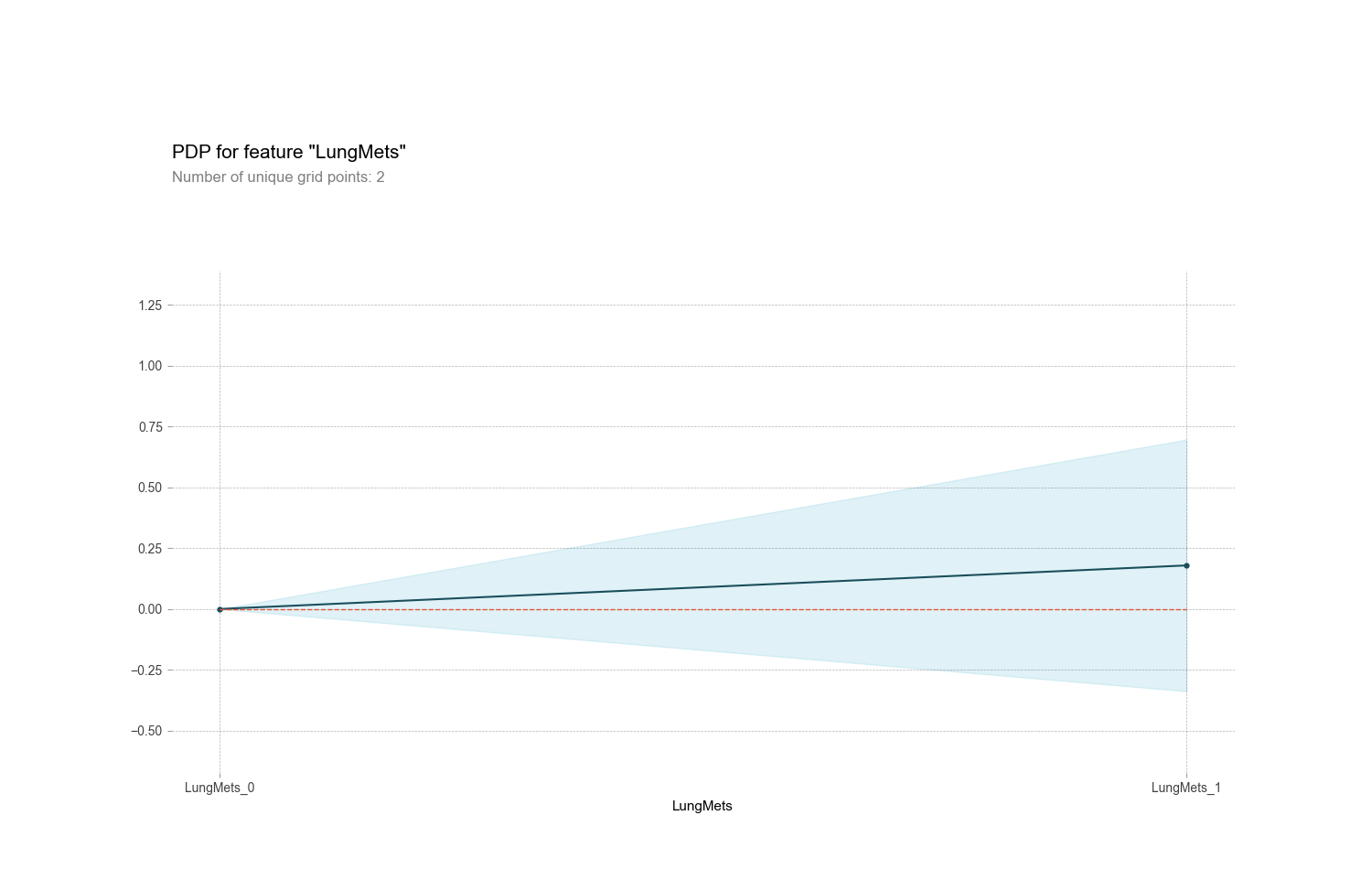
**

**Figure S3.** Partial dependent plots of clinical characteristics “lung metastasis”; the shaded portion represents the confidence interval.


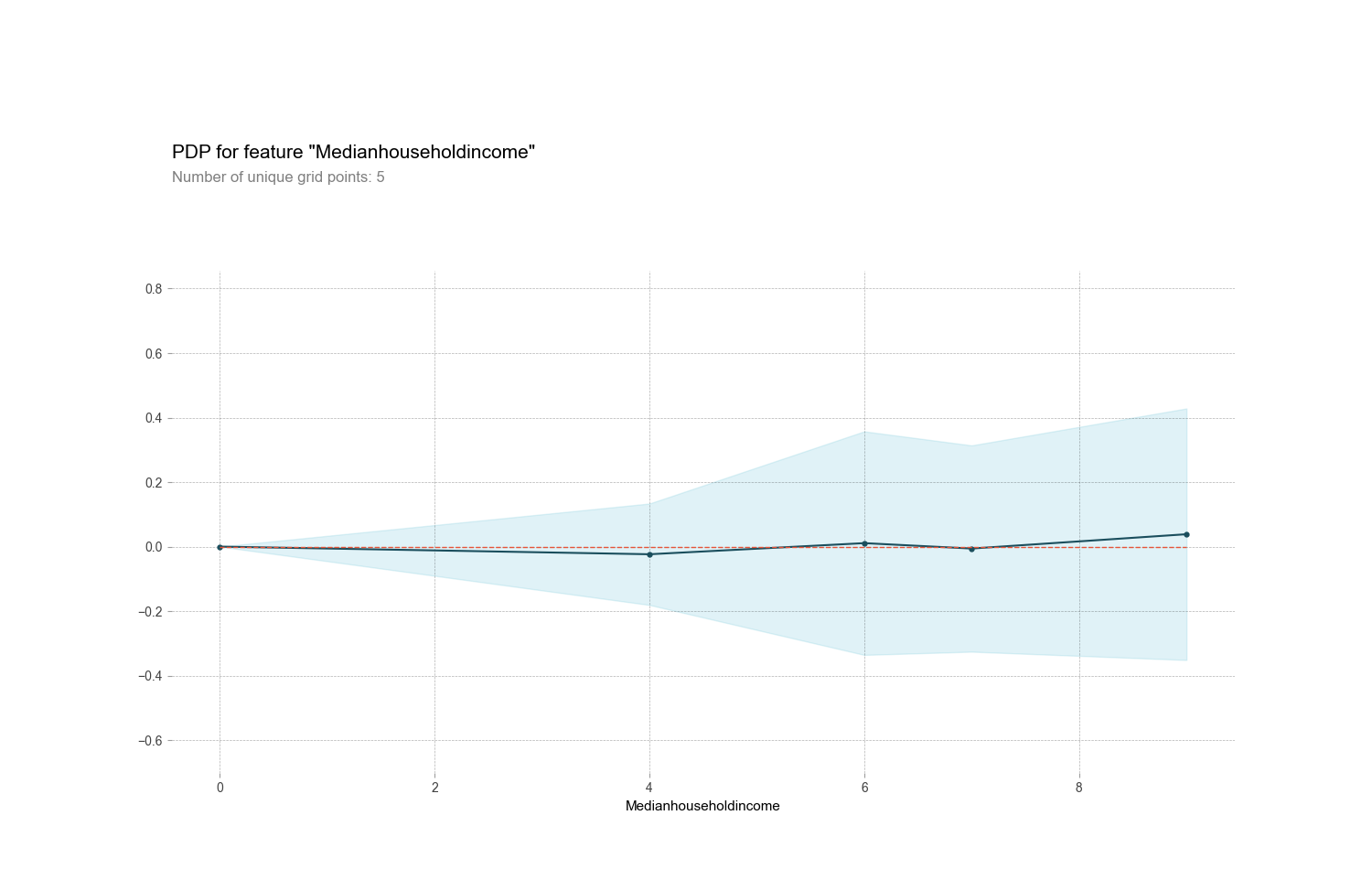


**Figure S4.** Partial dependent plots of clinical characteristics “median household income”; the shaded portion represents the confidence interval.


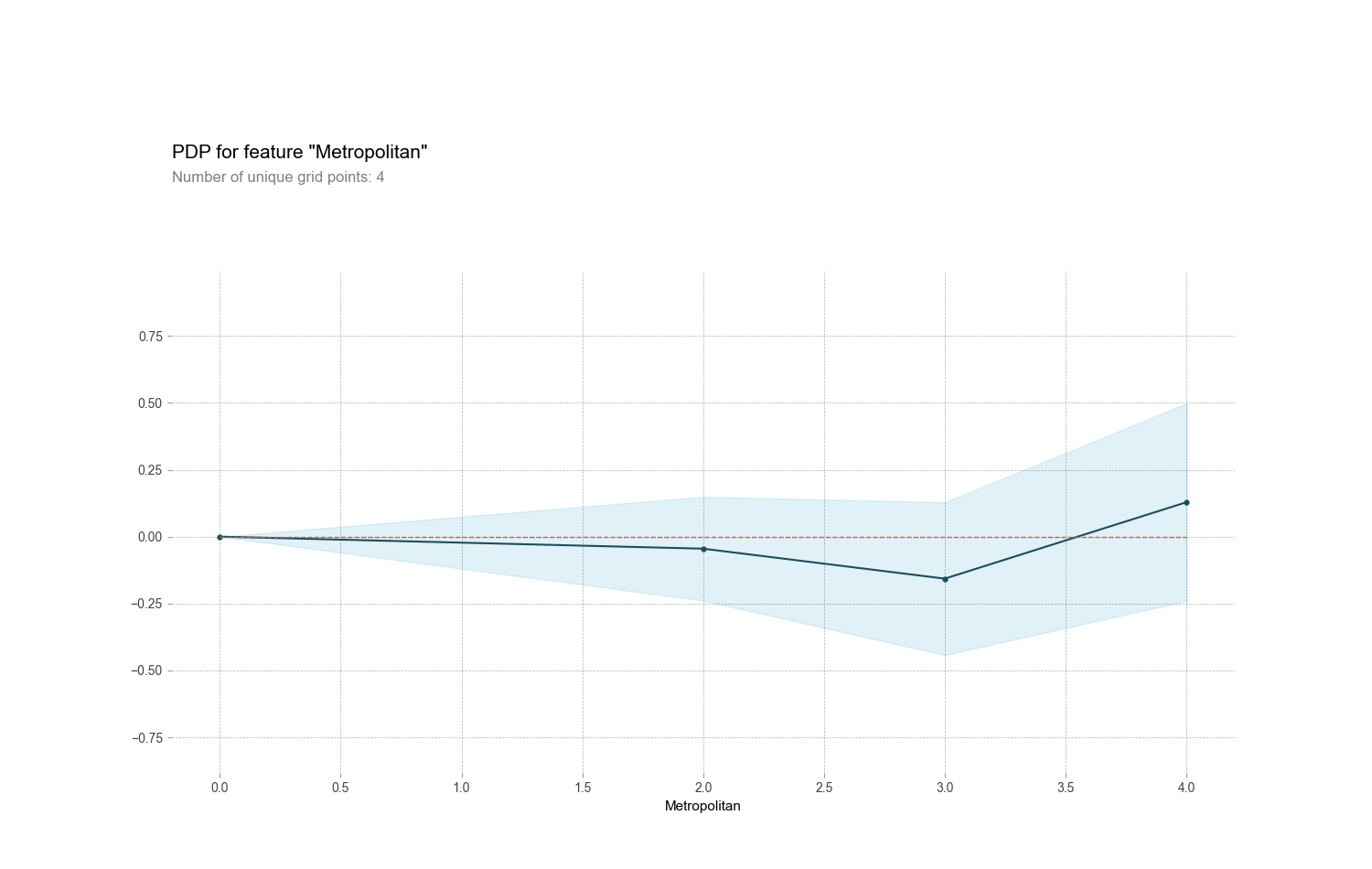


**Figure S5.** Partial dependent plots of clinical characteristics “metropolitan”; the shaded portion represents the confidence interval.


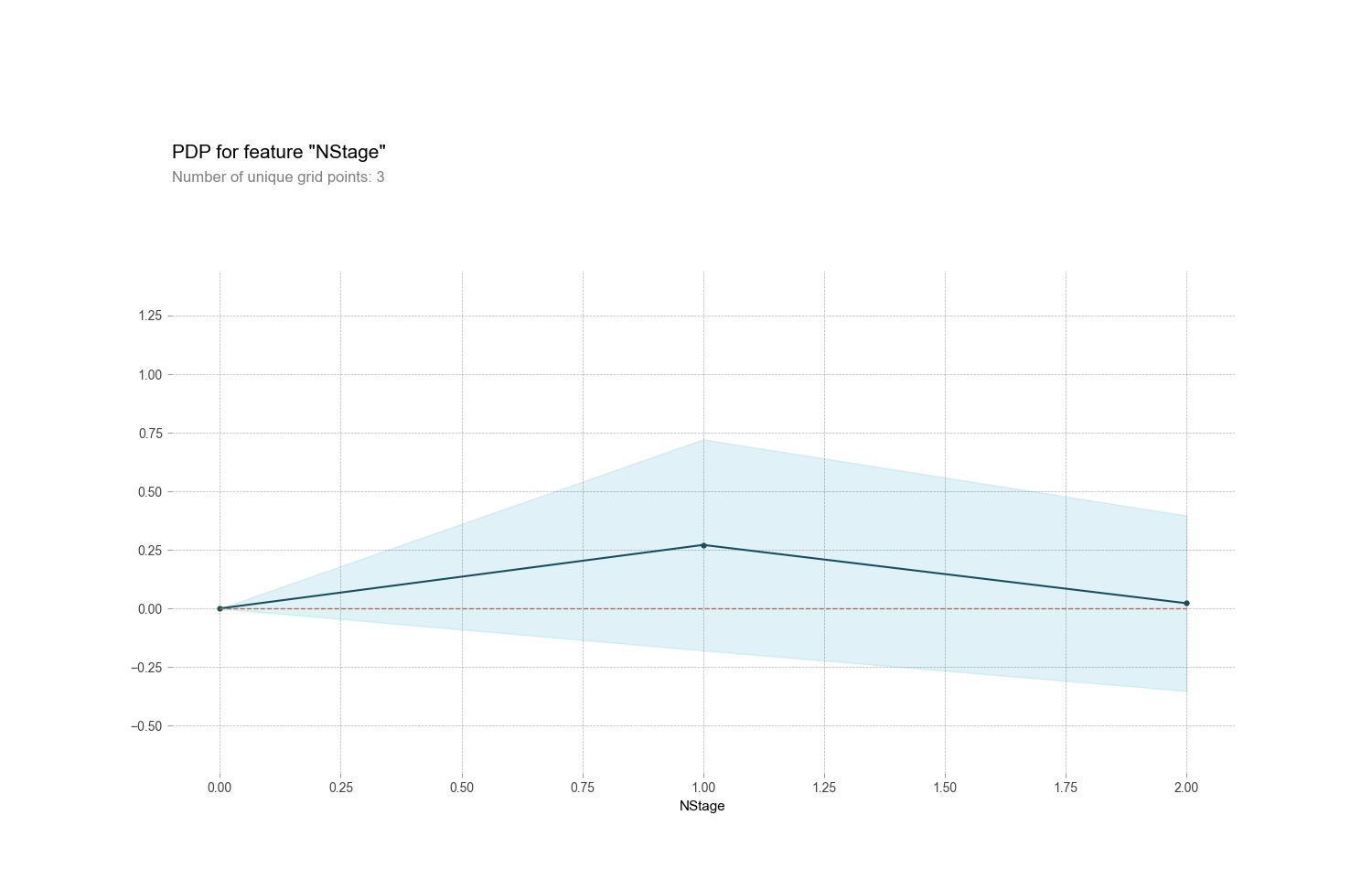


**Figure S6.** Partial dependent plots of clinical characteristics “N stage”; the shaded portion represents the confidence interval.


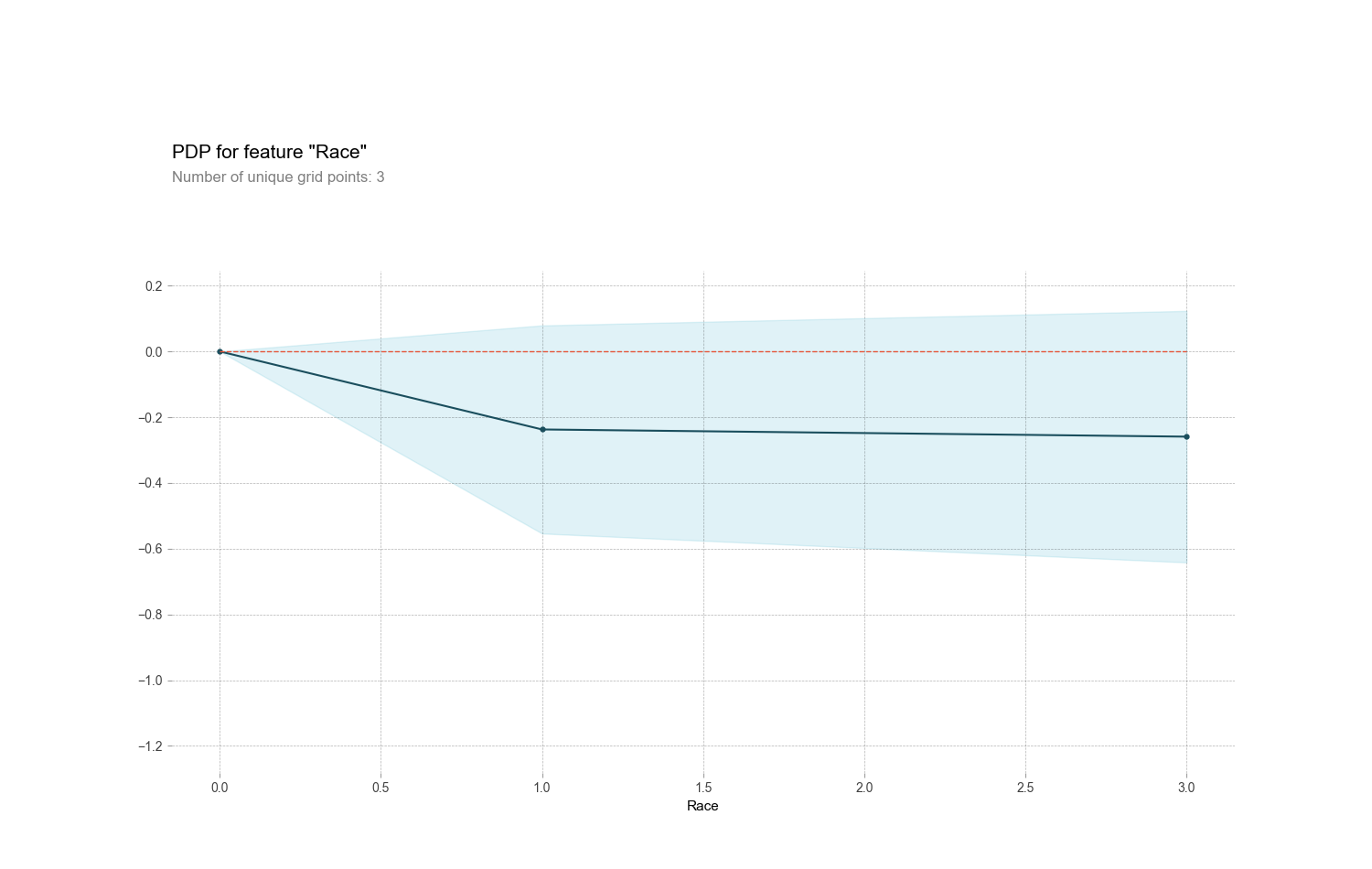


**Figure S7.** Partial dependent plots of clinical characteristics “race”; the shaded portion represents the confidence interval.

**Conflict of Interest, Funding Statement for production**

**Competing interests**

The authors declare that they have no competing interests.

**Funding**

This work was supported by the Taishan Scholar Project [grant number ts20190991], the Key R&D Project of Shandong Province [grant number 2022CXPT023] and the Scholar Project of Yantai’s “Double Hundreds plan”.
